# Long‐Term Health Effects of Football and Zumba Among Norwegian Female Hospital Employees: A 4‐Year Follow‐Up of a Cluster‐Randomized Trial

**DOI:** 10.1002/ejsc.12330

**Published:** 2025-06-07

**Authors:** Svein Barene, Peter Krustrup, Sigbjørn Litleskare, Andreas Holtermann

**Affiliations:** ^1^ Department of Public Health and Sport Science University of Inland Norway Elverum Norway; ^2^ Department of Sports Science and Clinical Biomechanics Sport and Health Sciences Cluster (SHSC) University of Southern Denmark Odense Denmark; ^3^ Danish Institute for Advanced Study (DIAS) University of Southern Denmark Odense Denmark; ^4^ Centre for Health and Technology University of South‐Eastern Norway Drammen Norway; ^5^ National Research Centre for the Working Environment Copenhagen Denmark

**Keywords:** aerobic dance, health promotion, health workers, long‐term effects, soccer, workplace

## Abstract

We evaluated the long‐term effects 4 years after a worksite exercise intervention among female hospital employees. In 2011, 107 female hospital employees were randomized into the two exercise groups, football and Zumba, offered 1–2 exercise sessions over 40 weeks, and a control group. Aerobic fitness, body composition, blood cholesterol, self‐reported job satisfaction, work‐related perceived physical exertion, and stress were measured at baseline, at the end of the intervention (40 weeks), and 4 years later. In this 4‐year follow‐up study, which consisted of 43 participants divided into a combined intervention group (*n* = 31) and controls (*n* = 12), we examined the long‐term effects after the intervention without continued support from the workplace or the research team. Compared with the controls, the intervention group had higher relative (*p* < 0.01) and absolute (*p* < 0.05) aerobic fitness, as well as higher power output at exhaustion during an incremental cycling test (*p* < 0.05). Furthermore, the intervention group had a higher job satisfaction (*p* < 0.05) compared to controls. On the contrary, the intervention group had less favorable results compared to controls related to perceived physical exertion at work (*p* < 0.05) and self‐reported stress (*p* < 0.05). Our study indicates that a worksite exercise intervention has long‐term beneficial effects on aerobic fitness, exercise capacity, and job satisfaction, although it might have side effects of higher work‐related physical exhaustion and stress. These findings provide valuable insight into the long‐term health effects of a worksite physical exercise intervention under real‐world conditions without continued project support.

**Summary**

- This study provides valuable insight into the long‐term effects of a worksite exercise intervention under real‐world conditions without project support.
- Beneficial aerobic fitness and exercise capacity achieved in the intervention group compared with controls during a 40‐week cluster‐randomized football and Zumba intervention was maintained 4 years later.
- Despite the intervention group maintaining significantly higher job satisfaction than the controls, they reported higher levels of work‐related physical perceived exertion and stress compared to the controls in the post‐intervention period up to 4 years later.

## 1 Introduction

Globally, the health care system is one of the most important institutions for ensuring the general population's physical, mental, and social health (Chengoden et al. 2023; Poon et al. 2022; Salyers et al. 2017), which underlines the importance of facilitating good working conditions and health among those working in health care. However, according to previous research, health care workers are daily exposed to work‐related factors such as high work demands, emotional stress, shift work, and understaffing (Eldin et al. 2021; Robertson et al. 2016; Petersen et al. 2023; Søvold et al. 2021). Ultimately, this can have a negative impact on the health of the workers, the recruitment of new employees, and the retention of qualified health care personnel (Broetje et al. 2020; Marufu et al. 2021).

Moreover, the combination of an aging population and the concurrent increase in people living with long‐term chronic conditions requires targeted measures for promoting the health of health care workers (Chengoden et al. 2023; Kyrarini et al. 2021). This challenge is further exacerbated by increasing difficulties related to recruitment and retention within the health care workforce (Alkan et al. 2024), often driven by burnout and occupational stress resulting from a sustained imbalance between physical and psychosocial job demands and employees' capacity to meet them (Lan et al. 2019).

Health‐promoting physical exercise programs at the workplace can potentially be such an effective targeted measure. A growing body of evidence, including systematic reviews and meta‐analyses, has demonstrated that participation in physical activity, whether through team‐based or individual activities, is associated with significant improvements in mental health and social functioning among adults (Eather et al. 2023; Koch et al. 2019). Specifically, interventions implemented in workplace settings have shown efficacy in reducing symptoms of occupational stress and psychological distress (Oliveira et al. 2023; Santos and Miragaia 2023; Shiri et al. 2023).

During the last decades, numerous worksite physical exercise interventions among health care workers have been implemented with varying results, both with regard to implementation, health effects, and sustainability (Burn et al. 2019; Conn et al. 2009; Muir et al. 2019; Mulchandani et al. 2019). However, previous interventions have mainly investigated short‐term effects, with < 11% of studies having follow‐up measurements more than 2 years after baseline (Mänttäri et al. 2020; Pohjonen and Ranta 2001; Vingård et al. 2009).

In a 40‐week randomized controlled worksite physical exercise intervention study among female health care workers that included football and Zumba, we found beneficial effects on aerobic fitness (Barene et al. 2013) and body composition (Barene, Krustrup, Brekke, et al. 2014), as well as job satisfaction (Barene et al. 2023). The aim of the current follow‐up study was to investigate the long‐term health effects of the exercise intervention in the 4‐year follow‐up period after the 40‐week intervention without continued project support from the workplace or the research team.

## 2 Methods

### 2.1 Study Design

This 40‐week randomized controlled exercise intervention, conducted among female hospital employees at a Norwegian hospital, was carried out from January to October 2011 and has been described in detail previously (Barene et al. 2013). The original study was ethically approved on 2010‐12‐13 by the Regional Committees for Medical and Health Research Ethics and further approved for a 4‐year follow‐up on 2014‐12‐18 based on the Health Research Act § 11 and the Research Ethics Act Section 4, Norway (2010/2385‐8). This study was registered in the International Standard Randomized Controlled Trial Number registry (ISRCTN61986892). The delay in publishing the data was due to resource and time constraints, as the team balanced competing academic and professional commitments following this study.

### 2.2 Recruitment of Participants and Randomization

The inclusion criteria for participation were hospital employees of either sex aged 25–65 years, with pregnancy, angina pectoris, and life‐threatening diseases as exclusion criteria. All enrolled employees met the inclusion criteria, as they were hospital staff aged 25–65 years. Apart from one employee who was excluded after the screening process due to pregnancy, none of the enrolled participants had angina pectoris or other life‐threatening conditions. Written informed consent was obtained from all participants. The recruitment process for the original intervention has been described in detail previously (Barene et al. 2013).

Briefly, a total of 118 hospital employees (of whom 107 were female; men were excluded from the statistical analyses due to their low numbers) were initially assigned to three large clusters. Cluster 1, consisting of personnel from a single department working in close proximity (*n* = 28), served as a reference for Cluster 2 (*n* = 27) and Cluster 3 (*n* = 29), which were matched by gender, BMI, age, and job seniority. The remaining participants were assigned to three smaller matched clusters: A (*n* = 11), B (*n* = 11), and C (*n* = 12).

Randomization was performed by blinded staff using a lot‐drawing procedure involving three boxes: (i) intervention groups (football, Zumba, control), (ii) large clusters (1–3), and (iii) small clusters (A–C). For each intervention group drawn from the box (i), one large cluster from the box (ii) and one small cluster from the box (iii) were drawn and assigned to that group. This process was repeated until all clusters had been allocated, resulting in the following group composition: football (Cluster 3 + C; *n* = 37), Zumba (Cluster 2 + B; *n* = 35), and control (Cluster 1 + A; *n* = 35).

**FIGURE 1:**
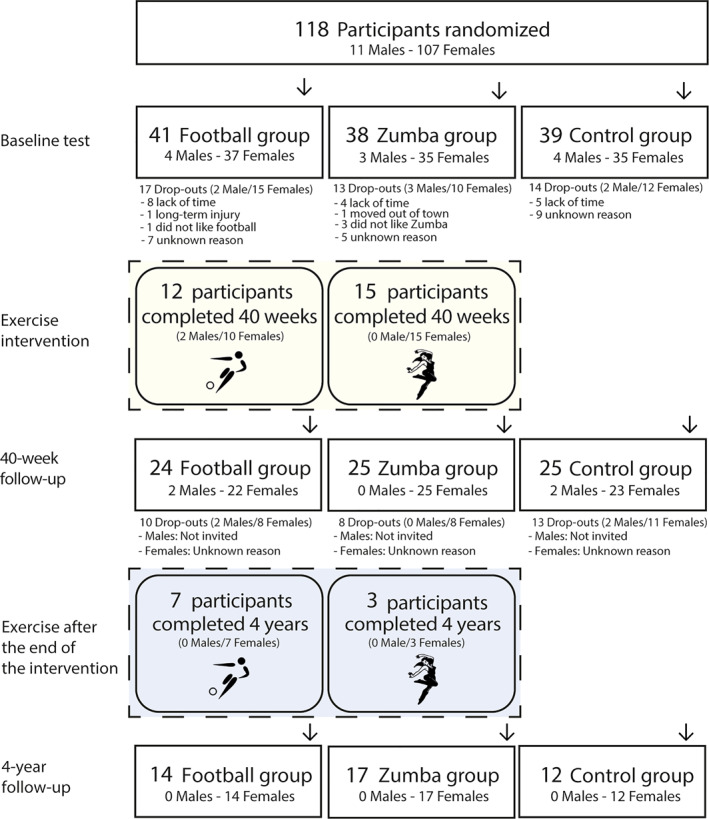
Flow chart of recruitment and adherence during the 40‐week intervention period, including the 4‐year follow‐up, with specification of the number of participants who completed.

Of these, 25 female employees (football, *n* = 10; Zumba, *n* = 15) completed the intervention up to the 40‐week follow‐up. An email invitation to participate in the 4‐year follow‐up was sent to those who had completed the 40‐week follow‐up 3 months beforehand. A total of 7 participants in the football group and 3 participants in the Zumba group completed the intervention activities up to the 4‐year follow‐up. Figure 1 provides a detailed flowchart with an overview of the number of participants throughout the 4‐year follow‐up.

### 2.3 Intervention Measurements Pre, During, and Post Intervention

Measurements were conducted at baseline and at the 4‐year follow‐up, including anthropometric characteristics, aerobic fitness (VO_2_peak assessed by incremental cycling test), body composition (measured by DXA scan), and fasting blood samples. Participants also completed an electronic survey (provided by questback.com), distributed anonymously via email to the participants. The survey contained 65 questions addressing background demographic information, work‐related factors such as job characteristics, overall job satisfaction, perceived physical exertion during work, perceived muscle pain and stress levels, sick leave days, and related topics. It took approximately 30–40 min to complete, and participants were encouraged to respond outside of working hours.

Short‐term effects on physiological health (Barene, Krustrup, Brekke et al. 2014; Barene et al. 2013), musculoskeletal pain (Barene, Krustrup, and Holtermann 2014), isometric strength and postural balance (Barene et al. 2016), work‐related health (Barene and Krustrup 2022), and workload functioning and sick leave (Barene et al. 2023) have been reported in previous publications.

Exercise participation and dropout rates in the two original intervention groups were monitored throughout the 40‐week intervention period. During the first 12 weeks, exercise participation in the Zumba group was recorded by the Zumba instructor, whereas participation in the football group was recorded by the project leader. After the 12‐week follow‐up, the project leader, in collaboration with the participants, created separate closed Facebook groups for each intervention group (football and Zumba). These groups allowed participants to self‐report their training participation online for the remainder of the intervention period, from Week 12–40.

Additionally, physical activity levels, defined as moderate‐to‐high‐intensity activity lasting > 20 min, were retrospectively recorded for both intervention groups and the control group during three distinct periods: (1) the 3 months preceding the intervention (October–December 2010), (2) the 40‐week intervention period (January–October 2011; supplementary physical activity for intervention group participants), and (3) the post‐intervention period extending to the 4‐year follow‐up (November 2011–May 2015).

### 2.4 Intervention Program

The design of the worksite‐initiated exercise intervention is previously described (Barene, Krustrup, Brekke et al. 2014). The intervention groups in this study were composed of colleagues from the same team/department. The intervention, which was organized through the workplace, was carried out in premises at or near the workplace outside working hours. During the first 12 weeks, both the football and the Zumba group were offered three 1‐h training sessions per week, with the opportunity for two 1‐h sessions during the last 28 weeks. Participants in both intervention groups had little or no previous experience with the exercises.

The football sessions were facilitated as small‐sided games in a traditional gymnastics hall (10 × 20 m) at the hospital, as well as in a municipal sports hall (20 × 40 m) located close to the hospital. The Zumba sessions were conducted at a fitness center close to the hospital and were supervised by certified instructors. Training sessions for both groups were scheduled outside of working hours, typically within 1 h after the end of the workday.

During the initial 12 weeks, participants in both intervention groups were offered five 1‐h sessions per week: three sessions held from 4:00 to 5:00 p.m. and two midday sessions to accommodate shift workers. For the subsequent 28 weeks, participants were expected to complete the training independently. To support this, training facilities were made available for both groups on two designated days per week from 4:00 to 5:00 p.m., as agreed upon with the participants, throughout the intervention period.

All participants received information about the results after the 40‐week follow‐up and were encouraged to continue with the exercise activities without further support from the workplace or the project management.

### 2.5 Statistical Analyses

All statistical analyses were performed using STATA version 18.0. Because of the relatively small number of participants in the football group (*n* = 14) and the Zumba group (*n* = 17) at the 4‐year follow‐up, combined with the absence of significant differences between these groups across any outcome measures, we decided to merge them into a single intervention group (*n* = 31) to increase the statistical power of the analyses. Potential selection bias from baseline to 4‐year follow‐up was assessed by two‐sample *t*‐tests on differences in baseline measures between those who dropped out during the 40‐week intervention period (*n* = 64) and completers of the 4‐year follow‐up (*n* = 43).

To test for a group (the intervention groups vs. the controls) by time (40 weeks and 4 years) interaction on our outcome measures, a linear mixed model analysis was conducted only on participants with measurements at baseline and 4‐year follow‐up. For the best model fit (Faraway 2016), all analyses were adjusted for baseline values and job seniority, in addition to subject‐specific random effects. Restricted maximum likelihood with degrees of freedom based on the Satterthwaite approximation was used as the estimation method. The estimates for the difference obtained from the least square means, hereinafter referred to as the overall mean, form the basis for the effect measures on the outcome variables and the corresponding 95% confidence intervals and *p*‐values. *p* levels < 0.05 were accepted as statistically significant.

## 3 Results

To uncover potential selection bias from baseline to 4‐year follow‐up, groupwise comparisons of baseline measures for those who dropped out during the 40‐week intervention period (*n* = 64) versus completers of the 4‐year follow‐up (*n* = 43) were performed. Apart from higher perceived physical exertion (*p* < 0.05) and stress (*p* < 0.05) in the completers versus dropouts in the controls, no between‐group differences were observed for any outcome at baseline (Table 1).

**TABLE 1:** Baseline comparison of those who dropped out during the 40‐week intervention period (*n* = 64) versus completers of the 4‐year follow‐up (*n* = 43) in the intervention group and the controls, respectively.

Characteristics	Intervention group					Controls				
	Completers (*n* = 31)		Drop‐outs (*n* = 41)		Diff	Completers (*n* = 12)		Drop‐outs (*n* = 23)		Diff
	Mean	SD	Mean	SD	*p*‐value	Mean	SD	Mean	SD	*p*‐value
Age (years)	47.0	8.0	43.5	9.7	0.108	49.8	7.4	46.2	10.3	0.296
Job seniority (months)	79.8	60.1	88.3	65.5	0.582	81.7	86.5	61.0	51.3	0.385
Body weight (kg)	70.2	9.8	70.2	7.9	0.992	73.8	10.7	70.2	11.9	0.392
Body mass index (kg/m^2^)	25.1	3.1	25.0	2.7	0.889	27.1	3.8	25.3	3.4	0.164
Systolic blood pressure (mmHg)	111.8	12.9	114.3	18.6	0.527	115.0	12.6	113.0	13.1	0.664
Diastolic blood pressure (mmHg)	72.2	7.4	72.1	10.5	0.989	74.3	8.9	72.6	8.6	0.577
VO_2_peak (mL/kg/min)	32.8	6.0	31.9	6.2	0.514	32.2	5.6	33.0	7.5	0.761
VO_2_peak (L/min)	2.27	0.32	2.22	0.43	0.575	2.34	0.37	2.24	0.29	0.393
Power output at exhaustion (W)	230	35	214	44	0.098	220	36	223	31	0.766
Time to exhaustion (s)	336	46	318	51	0.136	317	52	330	39	0.385
Total body fat mass (kg)	23.0	6.5	23.1	5.7	0.946	25.4	6.8	23.9	8.0	0.587
Total body fat percentage (%)	35.0	6.5	35.6	5.5	0.694	36.7	4.9	36.1	7.2	0.778
Total body lean mass (kg)	41.7	5.0	41.3	4.4	0.728	42.8	4.5	40.7	4.5	0.204
S‐cholesterol (mmol/L)	5.4	1.2	5.1	0.8	0.181	5.7	1.0	5.6	1.1	0.838
Job satisfaction (1–4)	3.5	0.5	3.4	0.5	0.576	3.5	0.5	3.7	0.5	0.127
Perceived physical exertion (1–15)	5.8	3.8	5.7	3.4	0.942	5.5	2.5	3.3	2.8	0.038
Perceived stress level (0–5)	1.9	0.6	1.9	0.7	0.735	2.0	0.9	1.5	0.6	0.045

*Note:* Data are presented as means ± SD. The *p*‐values refer to comparisons between all groups.

### 3.1 Characteristics of the Population at 4‐Year Follow‐Up

At the 4‐year follow‐up (*n* = 43), the average age, job seniority, and body mass index (BMI) of participants were 51.7 ± 7.8 years, 134.3 ± 67.4 months, and 24.7 ± 3.0 kg/m^2^, respectively. No significant between‐group differences were observed for any of the included demographic or outcome variables (Table 2). Regarding current employment status, 19 participants in the intervention group and 9 in the controls remained in the same professional position. Eleven participants in the intervention group and 1 in the controls had taken new positions in other departments, whereas 1 participant from the intervention group had changed workplace. Additionally, 1 participant in the intervention group and 2 in the controls had retired.

**TABLE 2:** Four‐year characteristics of age, job seniority, anthropometry, body mass index (BMI), peak oxygen uptake (VO_2_peak), time to exhaustion, and power output during an incremental bicycle test, total body fat mass and percentage, total body lean mass, S‐cholesterol, job satisfaction, perceived physical exertion at work (RPE), and work‐related stress for the intervention group (*n* = 31) and the controls (*n* = 12), respectively.

Characteristics	Intervention group		Controls		Total		Diff
	Mean	SD	Mean	SD	Mean	SD	*p*‐value
Age (years)	51.0	8.0	53.8	7.4	51.7	7.8	0.302
Job seniority (months)	133.8	60.1	135.7	86.5	134.3	67.4	0.937
Height (cm)	167.4	5.7	165.6	7.3	166.9	6.1	0.384
Body weight (kg)	68.0	8.7	70.8	11.5	68.8	9.5	0.405
Body mass index (kg/m^2^)	24.3	2.7	25.7	3.3	24.7	2.9	0.155
Systolic blood pressure (mmHg)	126.6	21.9	133.2	16.7	128.5	20.6	0.357
Diastolic blood pressure (mmHg)	76.1	11.3	79.3	7.3	77.0	10.3	0.370
VO_2_peak (mL/kg/min)	30.5	5.4	28.6	5.4	29.9	5.4	0.309
VO_2_peak (L/min)	2.08	0.29	2.02	0.34	2.06	0.30	0.556
Power output at exhaustion (W)	231	35	212	41	226	38	0.128
Time to exhaustion (s)	253	43	231	47	247	45	0.156
Total body fat mass (kg)	21.7	5.8	24.1	7.0	22.3	6.2	0.255
Total body fat percentage (%)	33.5	5.8	35.5	4.9	34.0	5.6	0.304
Total body lean mass (kg)	42.2	4.6	42.9	5.2	42.4	4.7	0.660
S‐cholesterol (mmol/L)	5.5	0.9	5.9	1.0	5.6	0.9	0.200
Job satisfaction (1–4)	3.4	0.6	3.1	0.3	3.4	0.5	0.112
Perceived physical exertion (1–15)	6.7	3.4	5.3	3.3	6.3	3.4	0.316
Perceived stress level (0–5)	1.9	0.8	1.7	1.0	1.9	0.8	0.573

*Note:* Data are presented as means ± SD. The *p*‐values refer to comparisons between all groups.

At the 4‐year follow‐up, 39 participants (90.70%; 28 in the intervention group and 11 in the controls) completed the electronic questionnaire. Adherence to the original intervention activities varied among participants who completed the questionnaire (*n* = 28) after the 40‐week intervention period. Ten participants (35.71%) continued the intervention activity up to the 4‐year follow‐up (Figure 1), whereas 11 participants (39.29%) discontinued within the first year after the intervention ended. The remaining 10 participants (35.71%) stopped participating in the intervention activity shortly after the 40‐week follow‐up.

Among the 39 respondents, 23 participants in the intervention group (82.14%) maintained a physical activity frequency of 2–3 sessions or more per week between the 40‐week follow‐up and the 4‐year follow‐up, compared to 6 participants (54.55%) in the controls. Furthermore, 25 participants in the intervention group (89.29%) and 6 participants in the controls (54.55%) reported that their participation in the research project had increased their motivation for a physically active lifestyle.

Table 3 provides an overview of the average training frequency across the 4‐year period, defined as the number of weekly moderate‐to‐high‐intensity sessions lasting > 20 min. Retrospectively recorded physical activity habits identified the most commonly performed activities in both the intervention group and the controls during three distinct periods: pre‐intervention, during the intervention (including supplementary activities for intervention group participants), and post‐intervention. The most frequently performed activities, ranked by prevalence, were brisk walking, cycling (commuting to and from work), strength training, running, skiing, and aerobics.

**TABLE 3:** Average training frequency (weekly sessions of moderate‐to‐high‐intensity physical activity lasting > 20 min) pre‐, during, and post‐40‐week intervention period through to the 4‐year follow‐up for the intervention group (*n* = 28) and the control group (*n* = 11).

Physical activity habits	The intervention group		The control group		Diff
	Mean	SD	Mean	SD	*p*‐value
Pre‐intervention	1.8	0.8	1.7	0.6	0.557
During the intervention					
Baseline to 12‐week follow‐up	2.9	0.7	1.6	0.8	0.000
12–40 weeks follow‐up	2.1	1.0	1.7	0.8	0.245
Post‐intervention	2.2	0.8	1.7	0.6	0.056

*Note:* Data are presented as means ± SD. The *p*‐values refer to comparisons between all groups.

### 3.2 Long‐Term Fitness and Health Effects

**FIGURE 2:**
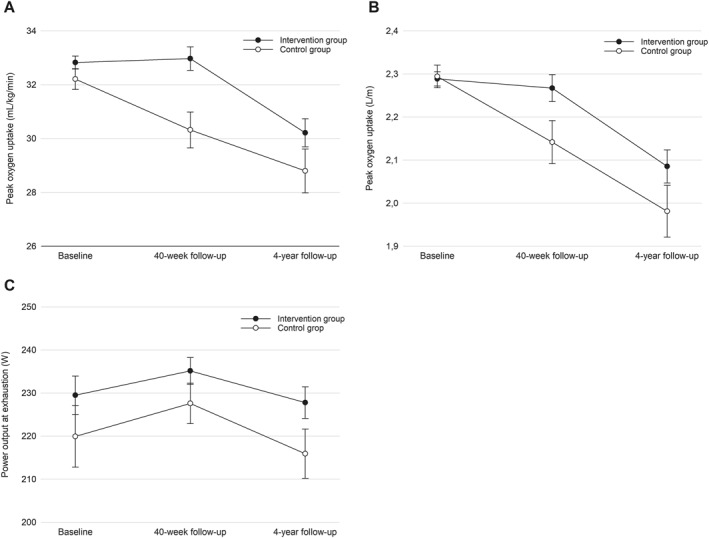
Between‐group differences throughout the intervention period and 4‐year follow‐up in relative (A) and absolute (B) VO_2_peak, as well as in power output at exhaustion (C).

In terms of aerobic fitness, the intervention group showed significantly higher relative VO_2_peak (2.1 mL/kg/min, 95% CI 0.6–3.6, *p* < 0.01) and absolute VO_2_peak (0.11 L/min, 95% CI 0.00–0.23, *p* < 0.05) from baseline to 4‐year follow‐up compared to the controls. Moreover, the intervention group had a significantly higher power output at exhaustion in the incremental cycling test (10 W, 95% CI 0.2–19.3, *p* < 0.05) than the controls (Table 4, Figure 2A–C). No further significant between‐group differences were observed for the other fitness and health outcomes (Table 4).

**TABLE 4:** Within‐group changes from baseline to 4‐year follow‐up in the intervention group and the controls, respectively, as well as between‐group differences based on least mean squares.

Outcome measure	Time	Intervention group (*n* = 31)				Controls (*n* = 12)				Between‐group differences		
		Mean	95% CI	Diff	*p*‐value	Mean	95% CI	Diff	*p*‐value	Diff	95% CI	*p*‐value
Body weight (kg)	40 week	69.3	68.1–70.5	0.4	0.545	71.3	69.4–73.2	2.7	0.022	−0.8	−2.9 to 1.2	0.427
	4 year	68.9	67.5–70.2			68.5	66.3–70.8					
Systolic blood pressure (mmHg)	40 week	111.1	108.1–114.1	17.8	0.000	109.4	104.9–113.8	21.8	0.000	−0.3	−5.4 to 4.7	0.901
	4 year	129.0	124.5–133.4			131.1	124.2–138.1					
Diastolic blood pressure (mmHg)	40 week	72.2	70.2–74.1	5.8	0.000	70.1	67.3–73.0	8.2	0.000	0.8	−2.4 to 4.1	0.612
	4 year	78.0	75.5–80.6			78.3	74.3–82.3					
VO_2_peak (mL/kg/min)	40 week	33.0	32.1–33.8	2.8	0.000	30.3	29.0–31.6	1.7	0.029	2.1	0.6–3.6	0.006
	4 year	30.2	29.2–31.2			28.6	27.1–30.2					
VO_2_peak (L/min)	40 week	2.27	2.21–2.33	0.18	0.000	2.14	2.04–2.24	0.16	0.003	0.11	0.00–0.23	0.045
	4 year	2.09	2.01–2.16			1.98	1.86–2.10					
Power output at exhaustion (W)	40 week	235	229–241	7	0.090	228	218–237	12	0.078	10	0.2–19.3	0.045
	4 year	228	221–235			216	205–227					
Time to exhaustion (s)	40 week	343	334–352	95	0.000	337	324–351	98	0.000	7	−7 to 21	0.336
	4 year	248	238–258			239	224–255					
Total body fat mass (kg)	40 week	2.3	2.2–2.4	0.6	0.223	2.4	2.3–2.5	1.7	0.042	0.7	−2.2 to 0.8	0.355
	4 year	2.2	2.1–2.3			2.3	2.1–2.4					
Total body fat (%)	40 week	34.7	33.8–35.5	0.7	0.154	36.6	35.3–37.8	2.2	0.007	−1.2	−2.8 to 0.4	0.149
	4 year	33.9	32.8–35.0			34.4	32.6–36.2					
Total body lean mass (kg)	40 week	42.2	41.6–42.7	0.3	0.255	41.2	40.4–42.0	1.0	0.024	0.6	−0.3 to 1.6	0.194
	4 year	42.5	41.9–43.1			42.2	41.1–43.2					
S‐cholesterol (mmol/L)	40 week	5.4	5.1–5.6	0.1	0.428	5.7	5.4–6.1	0.1	0.625	−0.3	−0.8 to 0.1	0.112
	4 year	5.5	5.2–5.8			5.8	5.4–6.2					
Job satisfaction (1–4)	40 week	3.5	3.3–2.7	0.7	0.601	3.3	3.0–3.6	0.3	0.194	0.3	0.0–0.6	0.033
	4 year	3.4	3.2–3.7			3.0	2.6–3.4					
Perceived physical exertion (1–15)	40 week	5.9	5.0–6.9	0.8	0.164	4.8	3.2–6.3	−0.1	0.914	1.6	0.1–3.2	0.039
	4 year	6.8	5.7–7.8			4.6	2.7–6.6					
Perceived stress level (0–5)	40 week	1.9	1.6–2.1	0.0	0.852	1.4	1.0–1.8	0.2	0.505	0.4	0.0–0.8	0.041
	4 year	1.9	1.7–2.2			1.6	1.1–2.1					

*Note:* Within‐group data are presented as mean change (95% CI), and between‐group data are presented as estimated overall mean difference (95% CI).

### 3.3 Changes in Work‐Related Factors

**FIGURE 3:**
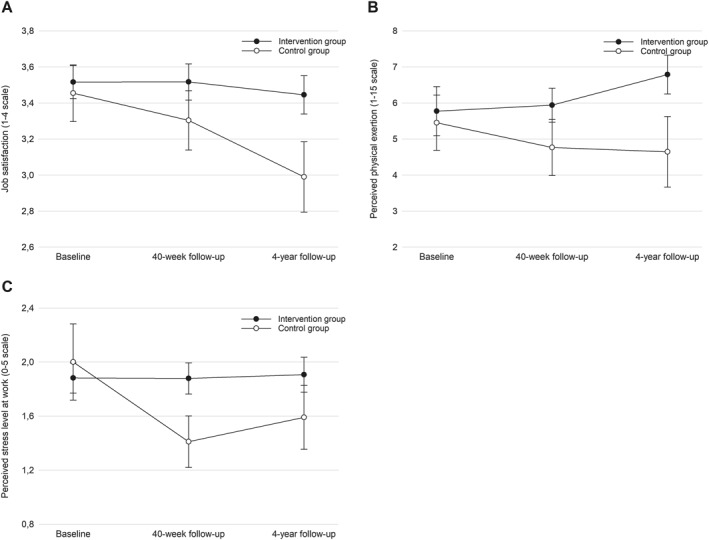
Between‐group differences throughout the intervention period and 4‐year follow‐up in job satisfaction (A), perceived physical exertion (B), and work‐related stress (C).

From baseline to 4‐year follow‐up, the intervention group showed significantly higher job satisfaction on a 1–4 scale (0.3 AU, 95% CI 0.0–0.6, *p* < 0.05) compared to the controls (Table 4, Figure 3A). However, the intervention group showed a less beneficial mean perceived physical exertion at work on a 1–15 scale (1.6 AU, 95% CI 0.1–3.2, *p* < 0.05) (Table 4, Figure 3B) and perceived stress related to work on a 0–5 scale (0.4 AU, 95% CI 0.0–0.8, *p* < 0.05) in comparison to the controls (Table 4, Figure 3C).

## 4 Discussion

The main findings of our study were that a 40‐week worksite exercise intervention without project support in the 4‐year follow‐up period had long‐term beneficial effects on aerobic fitness, exercise capacity, and job satisfaction, but also potential negative side effects on work‐related physical exhaustion and stress.

From baseline to 4‐year follow‐up, the intervention group maintained significantly higher aerobic fitness and power output at exhaustion in the incremental cycling test compared to the controls. Because we do not have valid measurements of exercise frequency during the 40‐week follow‐up, we can only speculate on possible reasons for the maintenance of the higher aerobic fitness and power output in the intervention group after 4 years. However, it is worth mentioning that a larger proportion in the intervention group compared to the controls reported that participation in this study led to increased motivation for physical activity, which was also supported by the fact that the intervention group reported to a greater extent being more physically active during the 4‐year follow‐up period.

Only one previous study has performed long‐term follow‐up measurements of aerobic fitness after a workplace exercise intervention among female home care workers (Pohjonen and Ranta 2001). In this study, significant intervention effects on aerobic fitness after a 9‐month training period were maintained at both 1‐ and 5‐year follow‐up compared to the control group, which is consistent with the findings of our study. Based on our knowledge, no previous randomized controlled worksite intervention studies have investigated long‐term effects on power output at exhaustion. This finding is therefore novel. This finding corresponds well with the improved aerobic fitness and report of more physical activity in the 4‐year follow‐up period among the intervention group than the controls. It remains uncertain whether these positive long‐term effects are exclusively due to our two intervention activities. However, it is plausible that activities with similar characteristics (such as team sports or dance‐based programs) could produce comparable benefits. We therefore encourage future studies to investigate this further.

In terms of total body fat mass and fat percentage, the significant between‐group differences at 40 weeks (directly after the workplace exercise intervention with project support) in favor of the intervention group were not maintained after 4 years. With the exception of a study by Pohjonen and Ranta (2001), which showed maintained between‐group differences in total fat percentage in favor of the intervention group at both 1‐ and 5‐year follow‐up, our findings are consistent with other studies with follow‐up periods longer than 1 year. These conflicting results related to long‐term effects on body fat should be followed up in future studies.

No significant between‐group difference was found in S‐cholesterol. Based on our knowledge, no previous randomized controlled worksite intervention studies have investigated long‐term effects on S‐cholesterol of a similar duration to our study. However, a previous 15‐month follow‐up of an 8‐week physical activity intervention delivered in the workplace also found that the improvements seen in total cholesterol at 8‐week follow‐up were not maintained at 15‐month follow‐up (Skogstad et al. 2018).

From baseline to 4‐year follow‐up, the intervention group showed significantly higher job satisfaction compared to the controls. A potential explanation for this could be that intervention participation created lasting positive social relationships that may have had an impact on the participants' perceived job satisfaction. Although previous meta‐analyses and overviews have shown examples that worksite physical exercise interventions can increase job satisfaction, it is important to emphasize that the majority of these are of shorter duration (< 1 year) (Conn et al. 2009; Shiri et al. 2023). Nevertheless, our study indicates that long‐term effects on job satisfaction can be achieved through this type of intervention.

Regarding the other work‐related factors, significant between‐group differences in disfavor of the intervention group compared to the controls were observed both for perceived physical exertion at work and stress. A possible explanation for these negative side effects observed in this study could be that unfavorable perceptions of the participants in the intervention group can be related to the withdrawal of the support from the workplace and the research group after 40 weeks. However, it is important to emphasize that this is not based on empirical data and should, therefore, be interpreted with caution. Further studies with repetitive measurements of adherence, physical activity, health, and work‐related outcomes throughout the follow‐up period are, therefore, needed to establish the knowledge needed.

### 4.1 Strengths and Limitations

Overall, this study uses a cluster‐randomized controlled design, which is considered a gold standard in the effectiveness evaluation of an intervention in a workplace setting. Another strength is that this study is one of the very few worksite physical exercise interventions internationally with long‐term follow‐up data up to 4 years after the end of the intervention. A weakness of the present long‐term follow‐up was the relatively large dropout (*n* = 27) between the 40‐week and 4‐year follow‐up (16 in the intervention group and 11 in the controls). In addition, we lacked information on the participants' weekly working hours throughout the 40‐week intervention, as these were only recorded in the pre‐intervention screening questionnaire. Another limitation is the absence of repeated measurements during the extended follow‐up period. However, conducting additional assessments could have increased participant burden and potentially influenced their behavior, thereby compromising the study's real‐world conditions. Furthermore, the use of self‐reported data linked to questions about work‐related psychosocial factors constitutes a potential weakness in this study.

In conclusion, our study indicates that a worksite exercise intervention has long‐term beneficial effects on aerobic fitness, exercise capacity, and job satisfaction, although it might have side effects of higher work‐related physical exhaustion and stress. These findings provide valuable insight into the long‐term health effects of a worksite physical exercise intervention under real‐world conditions without continued project support or resources.

## Conflicts of Interest

The authors declare no conflicts of interest.
